# Clinical features and predictors for disease natural progression in adults with Pompe disease: a nationwide prospective observational study

**DOI:** 10.1186/1750-1172-7-88

**Published:** 2012-11-12

**Authors:** Nadine AME van der Beek, Juna M de Vries, Marloes LC Hagemans, Wim CJ Hop, Marian A Kroos, John HJ Wokke, Marianne de Visser, Baziel GM van Engelen, Jan BM Kuks, Anneke J van der Kooi, Nicolette C Notermans, Karin G Faber, Jan JGM Verschuuren, Arnold JJ Reuser, Ans T van der Ploeg, Pieter A van Doorn

**Affiliations:** 1 Department of Neurology, Center for Lysosomal and Metabolic Diseases, Erasmus MC University Medical Center, ’s-Gravendijkwal 230, 3015 CE, Rotterdam, the Netherlands; 2 Department of Pediatrics, Center for Lysosomal and Metabolic Diseases, Erasmus MC – Sophia Children’s Hospital, University Medical Center, Rotterdam, the Netherlands; 3 Department of Epidemiology and Biostatistics, Erasmus MC University Medical Center, Rotterdam, the Netherlands; 4 Department of Clinical Genetics, Center for Lysosomal and Metabolic Diseases, Erasmus MC University Medical Center, Rotterdam, the Netherlands; 5 Department of Neurology, Rudolf Magnus Institute of Neurosciences, University Medical Center Utrecht, Utrecht, the Netherlands; 6 Department of Neurology, Academic Medical Center, Amsterdam, the Netherlands; 7 Department of Neurology, Radboud University Nijmegen Medical Center, Nijmegen, the Netherlands; 8 Department of Neurology, University Medical Center Groningen, Groningen, the Netherlands; 9 Department of Neurology, Maastricht University Medical Center, Maastricht, the Netherlands; 10 Department of Neurology, Leiden University Medical Center, Leiden, the Netherlands

**Keywords:** Acid α-glucosidase, Glycogen storage disease type II, OMIM number 232300, Lysosomal storage disorder, Disease progression, Natural course, Prognostic factors

## Abstract

**Background:**

Due partly to physicians’ unawareness, many adults with Pompe disease are diagnosed with great delay. Besides, it is not well known which factors influence the rate of disease progression, and thus disease outcome. We delineated the specific clinical features of Pompe disease in adults, and mapped out the distribution and severity of muscle weakness, and the sequence of involvement of the individual muscle groups. Furthermore, we defined the natural disease course and identified prognostic factors for disease progression.

**Methods:**

We conducted a single-center, prospective, observational study. Muscle strength (manual muscle testing, and hand-held dynamometry), muscle function (quick motor function test), and pulmonary function (forced vital capacity in sitting and supine positions) were assessed every 3–6 months and analyzed using repeated-measures ANOVA.

**Results:**

Between October 2004 and August 2009, 94 patients aged between 25 and 75 years were included in the study. Although skeletal muscle weakness was typically distributed in a limb-girdle pattern, many patients had unfamiliar features such as ptosis (23%), bulbar weakness (28%), and scapular winging (33%). During follow-up (average 1.6 years, range 0.5-4.2 years), skeletal muscle strength deteriorated significantly (mean declines of −1.3% point/year for manual muscle testing and of −2.6% points/year for hand-held dynamometry; both p<0.001). Longer disease duration (>15 years) and pulmonary involvement (forced vital capacity in sitting position <80%) at study entry predicted faster decline. On average, forced vital capacity in supine position deteriorated by 1.3% points per year (p=0.02). Decline in pulmonary function was consistent across subgroups. Ten percent of patients declined unexpectedly fast.

**Conclusions:**

Recognizing patterns of common and less familiar characteristics in adults with Pompe disease facilitates timely diagnosis. Longer disease duration and reduced pulmonary function stand out as predictors of rapid disease progression, and aid in deciding whether to initiate enzyme replacement therapy, or when.

## Background

Pompe disease is a rare autosomal recessive metabolic disorder, whereby mutations in the *GAA* gene lead to partial or total absence of the lysosomal enzyme acid α-glucosidase. The disease presents as a spectrum of phenotypes, ranging from a rapidly fatal phenotype in infants [1] to slower progressive phenotypes in older children and adults. [2,3] Many adults with Pompe disease are diagnosed late in life, when they are already in an advanced stage of the disease. While this may be due to clinicians’ unawareness, it may also be explained by similarities in clinical presentation with other ‘limb-girdle’ diseases such as the limb-girdle muscular dystrophies (LGMD), Becker muscular dystrophy, or metabolic myopathies. [4] Our first objective was to optimize future diagnosis in this patient group by classifying their specific clinical features, and by delineating the distribution and severity of muscle weakness and the sequential involvement of individual muscle groups during the course of the disease.

Since 2006, enzyme replacement therapy (ERT) with recombinant human acid α-glucosidase has been approved for the treatment of Pompe disease. In infants, treatment generally improves cardiorespiratory function and motor function, and prolongs survival. [5-8] In older children and adults ERT was shown to improve or stabilize skeletal muscle strength, muscle function and respiratory function. However, the magnitude of the therapeutic response varies between individual patients. [9-14] To fully assess the effects of enzyme therapy, and to decide whom to treat and when to start treatment, it is necessary to be optimally informed about the course of disease prior to treatment, and about factors influencing disease progression. We thus prospectively studied the natural disease course, and aimed to identify prognostic factors for faster disease progression and poor outcome in a large cohort of adult Pompe patients.

## Methods

### Participants and study design

We performed a single-center, prospective, cohort study, in which participation was open to all adults diagnosed with Pompe disease who had not yet received treatment with enzyme replacement therapy. Their diagnosis was confirmed by acid α-glucosidase assay in leukocytes or fibroblasts and by mutation analysis. All patients were seen between October 2004 and August 2009 at Erasmus MC University Medical Center, the designated center of expertise for Pompe disease in the Netherlands. The interval between visits was three to six months. Patients were recruited either through neuromuscular centers in the Netherlands and Belgium, through the Dutch neuromuscular patient organization, or were referred to our Center by their treating physicians. The research protocol was approved by the Central Committee on Research Involving Human Subjects in the Netherlands (CCMO). All patients provided written informed consent.

Seven patients participated in the placebo arm of the randomized, placebo controlled trial on the safety and efficacy of alglucosidase alfa in late-onset Pompe disease. [13] Data on these patients collected during this period are included in the present analyses. We have previously reported long-term retrospective data on muscle strength and pulmonary function in 16 patients with Pompe disease. [15] While 12 of these patients participated in the current study, the present analyses were based solely on new, prospectively obtained data.

### Procedures

We gathered information on the following: 1) the nature of first symptoms and the age at which these had presented; 2) the age at which the diagnosis had been made; 3) duration of disease since onset of first symptoms; 4) the presence of specific clinical features such as scoliosis, bulbar involvement (defined as weakness of muscles involved in speech, chewing and swallowing), winging of the scapula (defined as a clearly visible protrusion of the scapula when the patient was in a resting position or was lifting the arms anteriorly or sidewards), muscle atrophy, or ptosis; 5) the use of a wheelchair or walking aids; 6) skeletal muscle strength; 7) the use of mechanical ventilatory support; 8) the number of hours of ventilatory support per day; 9) pulmonary function; 10) cardiac function; 11) acid α-glucosidase activity in leukocytes and fibroblasts; 12) serum creatine kinase (CK); and 13) type of *GAA* mutation.

#### Skeletal muscle strength and muscle function

By manual muscle testing using the Medical Research Council (MRC) grading scale [16] (range 0–5; all patients were examined by NvdB or JdV), we measured 25 different muscle groups throughout the body to define the distribution of skeletal muscle weakness and the severity of involvement of the separate muscle groups. We calculated a sumscore (range 0–130) for the muscle groups that were involved most: neck extensors, neck flexors, and bilateral shoulder adduc-tors, shoulder abductors, shoulder exorotators, shoulder endorotators, elbow flexors, elbow extensors, hip extensors, hip flexors, hip abductors, hip adductors, knee flexors and knee extensors. This score was subsequently converted to a percentage of the maximum possible score. Although the abdominal muscles and trunk muscles were frequently involved, we did not include these muscle groups in the MRC sumscore since they were difficult to grade.

Hand-held dynamometry (HHD) (Cytec dynamometer, Groningen, the Netherlands) was used as a second measure of muscle strength to examine the following muscle groups: neck extensors, neck flexors and bilateral shoulder abductors, elbow flexors, elbow extensors, hip flexors, hip abductors, knee flexors and knee extensors. The value (Newton) measured in each muscle group was expressed as a percentage of the median strength of healthy females and males [17], and then combined into a sumscore by averaging these for all 16 muscle groups, producing a score between 0 and 100 percent.

Muscle function was assessed using the Quick Motor Function Test (QMFT). [18] A total score (range 0–64) was obtained by adding the scores of all items. This was then expressed as a percentage of the maximum score.

#### Pulmonary function

Forced vital capacity (FVC) was measured using a Lilly type pneumograph (Viasys Healthcare, Würzburg, Germany) or the KoKo spirometry system (Ferraris Respiratory, Louisville, USA) with the patient in upright seated and supine positions, according to ATS/ERS standards. [19] Results were expressed as a percentage of predicted normal values. [20] A measured value below 80% of the predicted value was considered to be abnormally low. Seven male patients who were invasively ventilated were artificially allotted a FVC value of 10% – just below the least observed value – since their omission might have led to biased results. These seven patients were however excluded from the longitudinal analysis.

### Statistical analysis

Baseline characteristics are summarized using descriptive statistics. Differences between males and females, and between patients with and without scapular winging, were assessed using χ^2^ tests (wheelchair use, use of mechanical ventilation, and presence of scoliosis, bulbar muscle weakness, scapular winging or ptosis) or Mann–Whitney tests (strength of individual muscle groups, MRC sumscore, HHD sumscore, QMFT sumscore, and FVC measured in sitting and supine positions). We used the Spearman’s rank correlation coefficient (ρ) to calculate the relationships between residual enzyme activity and rate of decline in muscle strength and pulmonary function, and between serum CK activity and age, muscle atrophy and disease duration. Longitudinal analysis of muscle strength and pulmonary function was performed using repeated measures ANOVA (random coefficient models). The annual changes are expressed in absolute percentage points (pp/y). For subgroup analyses, patients were divided into groups on the basis of gender (male, female); wheelchair use (yes, no); use of mechanical ventilation (yes, no); age at first study visit (<50 or ≥50 years; taking the median as the cut-off point); disease duration (<15 or ≥15 years; taking the median as the cut-off point); MRC/HHD sumscore at study entry (categorization in tertiles); and FVC in sitting position at study entry (<80 or ≥80% predicted). Analyses were performed with SPSS for Windows (version 15, SPSS Inc., Chicago, IL) or SAS (version 9.1, SAS Institute Inc., Cary, NC). A p-value of ≤0.05 was considered statistically significant.

## Results

### Study population

In order of referral, we included 91 adult Pompe patients from the Netherlands – representing virtually all known patients in the Netherlands – and three patients from Belgium. On average, there was a seven-year delay between the first noted symptoms of Pompe disease and the actual diagnosis. The characteristics of the study population are summarized in Table 1.

**Table 1:** **Characteristics of the study population (n=94)**^**a**^

**General characteristics**	
Gender (males)	48 (51%)
Age at first study visit (years)	51.1 (38.3-60.6)
Age at onset of symptoms (years)	32.0 (25.5-40.0)
• < 18 years	10 (11%)
• ≥ 18 years	84 (89%)
Age at diagnosis (years)	40.2 (32.7-50.2)
Disease duration since onset of first symptoms at first study visit (years)	15.3 (7.7-24.7)
Time since diagnosis at first study visit (years)	9.2 (0.6-16.0)
• 0 to 5 years	41 (44%)
• 5 to 10 years	15 (16%)
• 10 to 15 years	10 (11%)
• > 15 years	28 (30%)
Use of walking aids	14 (15%)
Wheelchair use	30 (32%)
Age at start of wheelchair use (years)	49.0 (43–56)
Use of mechanical ventilation ^b^	27 (29%)
Age at start of mechanical ventilation (years)	48 (38.5-57.5)
**First symptoms noted**^**c**^	
Skeletal muscle weakness	93 (99%)
• Difficulty running	30 (32%)
• Difficulty performing sports	22 (23%)
• Difficulty climbing stairs	24 (26%)
• Difficulty walking	15 (16%)
• Difficulty rising from an armchair	11 (12%)
• Difficulty rising from a lying position	9 (10%)
Fatigue	17 (18%)
Muscle soreness / cramps	16 (17%)
Respiratory failure	1 (1%)
**Clinical features**	
Ptosis	22 (23%)
Bulbar muscle weakness ^d^	26 (28%)
Scapular winging ^d^	31 (33%)
Scoliosis	22 (23%)
Increased lumbar lordosis	62 (66%)
Prominent muscle atrophy	53 (56%)
• Shoulder girdle / upper arms	25 (27%)
• Trunk muscles	27 (29%)
• Pelvic girdle / Upper leg (Figure 1a)	40 (43%)
**Laboratory parameters**	
CK (U/l)	
• Males	449 (279–1040)
• Females	493 (237–715)
α-glucosidase activity in leukocytes (nmol glucose/h/mg protein) ^e^	1.2 (0.4-2.2)
α-glucosidase activity in fibroblasts (nmol 4-MU/h/mg protein) ^f^	13.0 (11.0-15.0)
Genotype	
• c.-32-13T>G / very severe or potentially less severe pathogenic mutation	92 (98%)
• c.671G>A / c.525del	1 (1%)
• unknown	1(1%)

4-MU: 4-methylumbelliferyl-α-D-glucopyranoside.

^a^ Data are number (%) or median (IQR). ^b^ More men then women used mechanical ventilation (p=0.009). ^c^ Two or more complaints were counted if these occurred within the same year. ^d^ More men than women had scapular winging and bulbar muscle weakness (p=0.001 and p=0.05). ^e^ control range 48 to 215 nmol glucose/h/mg protein. ^f^ control range 45 to 180 nmol 4-MU/h/mg protein.

### Baseline measurements

#### Characteristic clinical features

**Figure 1:**
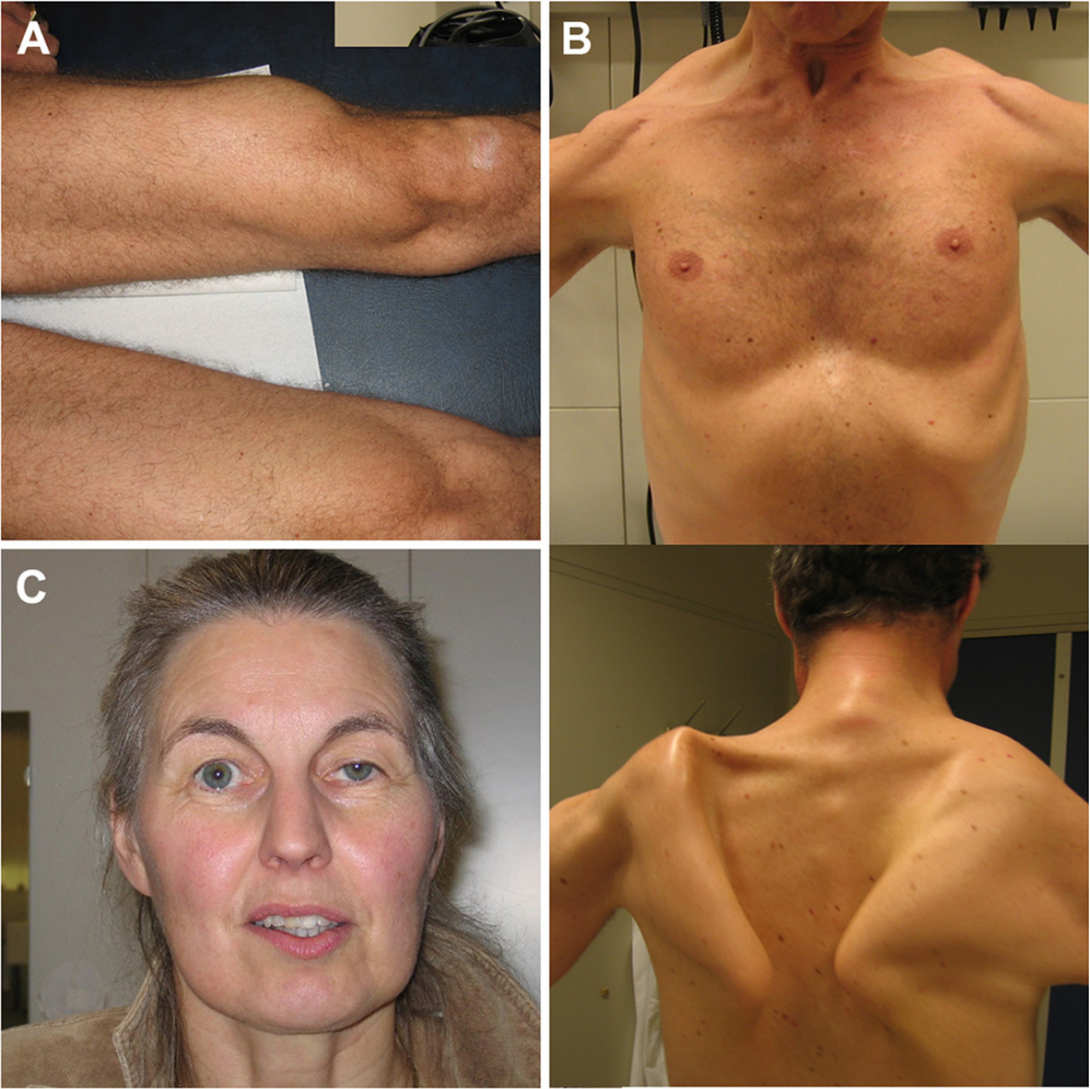
**Clinical features in Pompe disease.** Atrophy of the quadriceps muscle (**A**), scapular winging (**B**), and ptosis (**C**) as notable clinical features in adults with Pompe disease. Photographs are printed with permission of the patients.

A substantial number of patients had less familiar features of Pompe disease, such as bulbar muscle weakness (28%), prominent scapular winging (33%, Figure 1b), or ptosis – not accompanied by external eye-movement disturbances (23%; Figure 1c). Seventy-one percent of patients with scapular winging had bulbar muscle weakness, against 37% without scapular winging (p=0.001).

#### Clinical distribution and severity of muscle weakness, and sequence of muscle involvement during the disease course

**Figure 2:**
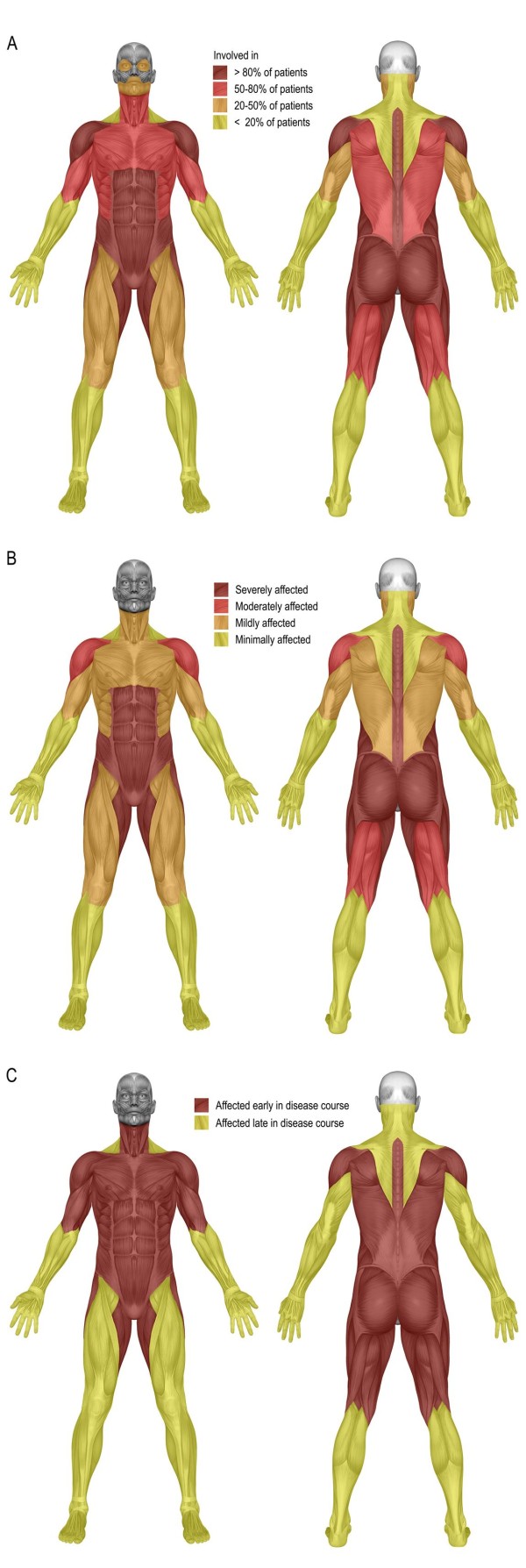
**Muscle weakness in adults with Pompe disease.** Distribution of skeletal muscle weakness (**A**), severity of muscle weakness of the individual muscle groups (**B**), and involvement of the individual muscles over time (**C**) in 94 adults with Pompe disease.

Shoulder abductors, abdominal muscles, paraspinal muscles, hip flexors, hip extensors, hip adductors, and hip abductors were affected in more than 80% of all patients (Figure 2a). The strength of the quadriceps muscle was reduced in only 55% of patients. The muscles of the hands and feet were relatively spared, being affected in under 10% of patients. Abdominal muscles, paraspinal muscles – with exception of the neck extensors and neck flexors –, hip flexors, hip extensors, hip adductors and hip abductors were the most severely affected muscles (Figure 2b). The pattern of muscle weakness was symmetrical in 95% of patients, and the distribution of weakness did not differ between males and females. Relative to patients without scapular winging, patients with scapular winging had more severe involvement of the shoulder girdle musculature (trapezius muscle, deltoid muscle, pectoral muscle, shoulder exorotators, and shoulder endorotators; all p<0.02). The ‘limb-girdle’ and trunk muscles were affected early in the course of the disease, while the distal muscle groups – if they were involved at all – were affected late in the course of the disease (Figure 2c).

#### Cardiac evaluation

In a subset of 51 patients cardiac evaluation was performed, comprising electrocardiography, Holter monitoring, two-dimensional echocardiography, low-dose dobutamine stress echocardiography, and tissue Doppler imaging. One patient had a mild hypertrophic cardiomyopathy, while four other patients had minor cardiac abnormalities that could be attributed to advanced age, hypertension or preexisting cardiac pathology unrelated to Pompe disease [21,22].

### *Laboratory parameters*

Most patients had moderately increased serum CK; in two patients it was more than 10 times the upper limit of normal, while 10 had a normal serum CK. Serum CK activity was moderately inversely associated with age (*ρ*=−0.71, p<0.001), disease duration (*ρ*=−0.45, p<0.001), and the presence of muscle atrophy (*ρ*=−0.53, p<0.001).

### Prospective follow-up

#### General aspects

Prospective follow-up data for a period longer than six months were available for 66 patients (median 1.6 years, range 0.5-4.2 years), 52 of whom were followed for longer than one year, and 22 of whom for longer than two years. Within the follow-up period, one patient became wheelchair bound, mechanical ventilation was initiated in four patients, and eight patients needed to increase their number of hours of ventilation per day. One severely affected patient died due to pneumonia complicated by respiratory failure.

#### Disease progression and predictors for disease outcome

##### Muscle strength and muscle function

**Figure 3:**
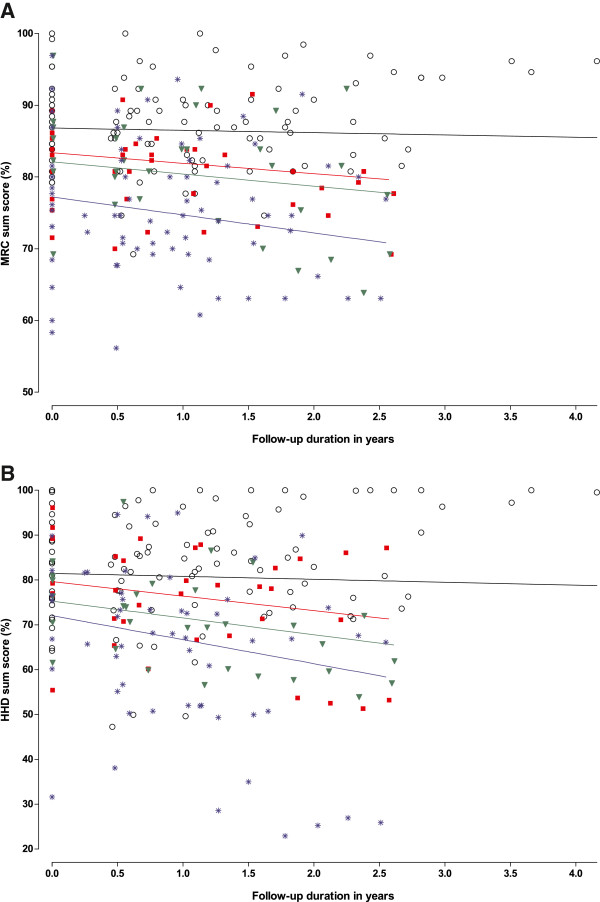
**Longitudinal changes in muscle strength.** Rate of disease progression measured by manual muscle testing (MRC sumscore) (**A**) and hand-held dynamometry (HHD sumscore) (**B**) related to follow-up duration measured from time of inclusion in the study for 66 adults with Pompe disease. The figure shows the measured values and regression lines at group level for the following subgroups: 1) Patients with normal pulmonary function (FVC ≥80% predicted) and disease duration <15 years (circles, black line); 2) patients with normal pulmonary function (FVC ≥80% predicted) and disease duration ≥ 15 years (red squares, red line); 3) patients with abnormal pulmonary function (FVC <80% predicted) and disease duration <15 years (green triangles, green line); and 4) patients with abnormal pulmonary function (FVC <80% predicted) and disease duration ≥ 15 years (blue asterisks, blue line).

**Figure 4:**
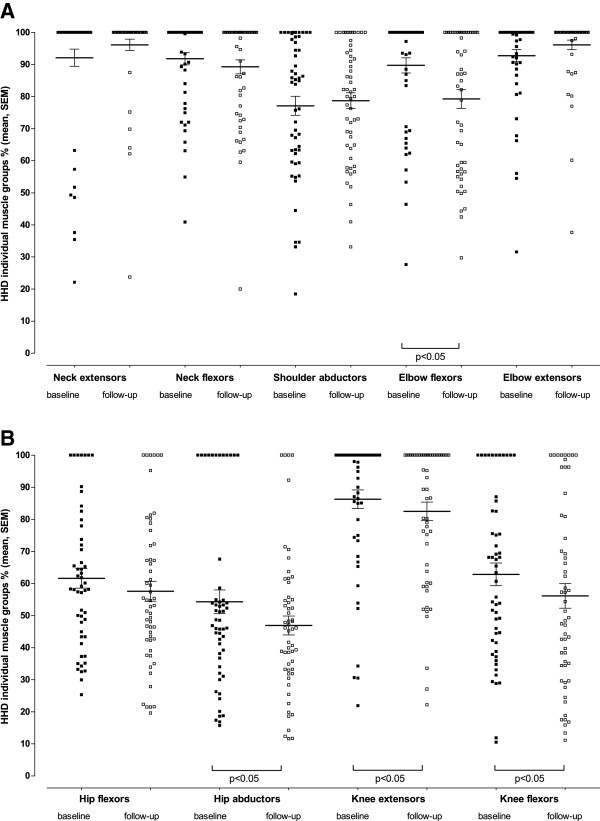
**Muscle strength in individual muscle groups measured by hand-held dynamometry.** Values for individual patients are shown at baseline (black squares) and during follow-up (last measured value) (open squares). Mean and standard error of the mean are given for each muscle group.

At baseline, the MRC sumscore ranged from 39.2% to 100% (median 84.7%, one patient had the maximum possible score). During follow-up, the MRC sumscore deteriorated by 1.3% points per year on average (p<0.001; Figure 3a). Baseline values for hand-held dynamometry ranged from 31.6% to 100% (median 77.0%, three patients had the maximum score). Within the observed follow-up period, HHD sumscore deteriorated by 2.6% points per year (p<0.001; Figure 3b). With regard to the individual muscle groups, strength declined significantly in the elbow flexors, hip abductors, knee extensors and knee flexors, ranging from -6.9 pp/y in elbow flexors to -2.5 pp/y in knee extensors (Figure 4). Subgroup analysis (Table 2) revealed that the decline was faster in patients with a reduced pulmonary function at baseline (FVC <80%) than in those with normal pulmonary function (−2.2 pp/y against −0.6 pp/y for MRC sumscore (p=0.01), and −4.5 pp/y against −1.4 pp/y for HHD sumscore (p<0.01)), and in patients who had had the disease for longer than 15 years compared to those who had been ill less long (−2.1 pp/y against −0.7pp/y for MRC sumscore (p=0.04), and −4.2 pp/y against −2.0 pp/y for HHD sumscore (p<0.01)). Baseline QMFT scores ranged from 16.7% to 100% (median 63.7%). The changes that were found in muscle strength were however not reflected in changes in the QMFT (annual change 0.05% points, p=0.9). In no subgroups was a significant decline observed.

**Table 2:** Disease progression in 66 adults with Pompe disease: annual changes in muscle strength and pulmonary function across various subgroups

	**MRC sumscore**			**HHD sumscore**			**QMFT score**			**FVC sitting position**			**FVC supine position**		
**Total group**	***n***	**Annual change (%/year)**	**95% CI**	***n***	**Annual change (%/year)**	**95% CI**	***n***	**Annual change (%/year)**	**95% CI**	***n***	**Annual change (%/year)**	**95% CI**	***n***	**Annual change (%/year)**	**95% CI**
	*65*	−1.30	−1.95 to −0.66	*55*	−2.6	−3.72 to −1.45	*62*	0.05	−0.76 to 0.86	*59*	−1.04	−2.14 to 0.06	*54*	−1.30	−2.42 to −0.19
**Subgroups**	***n***	**Annual change (%/year)**		***n***	**Annual change (%/year)**		***n***	**Annual change (%/year)**		***n***	**Annual change (%/year)**		***n***	**Annual change (%/year)**	
Gender															
• Female	*36*	−1.41		*31*	−2.97		*37*	−0.22		*36*	−1.38		*33*	−2.27	
• Male	*29*	−1.08		*24*	−1.99		*25*	0.57		*23*	−0.45		*21*	0.35	
Age at study entry ^a^															
• < 50 years	*34*	−1.39		*31*	−2.06		*33*	−0.17		*33*	−1.34		*31*	−2.22	
• ≥ 50 years	*31*	−1.21		*24*	−3.29		*29*	0.30		*26*	−0.69		*23*	−0.08	
Disease duration at study entry ^a^															
• < 15 years	*34*	**−0.70**^**c**^		*31*	**−1.95**^**e**^		*34*	−0.07		*35*	−1.36		*33*	−1.37	
• ≥ 15 years	*31*	**−2.09**^**c**^		*24*	**−4.18**^**e**^		*28*	0.17		*24*	−0.60		*21*	−1.16	
FVC (sitting) at study entry															
• ≥ 80% predicted	*33*	**−0.62**^**d**^		*32*	**−1.43**^**f**^		*33*	−0.21		*33*	−1.09		*31*	−1.59	
• < 80% predicted	*32*	**−2.21**^**d**^		*23*	**−4.52**^**f**^		*29*	0.31		*26*	−0.89		*23*	−0.92	
Ventilation at study entry															
• No	*49*	−1.12		*46*	−2.50		*50*	0.24		*50*	−1.30		*47*	−1.50	
• Yes	*16*	−1.83		*9*	−3.55		*12*	−0.70		*9*	1.07		*7*	0.32	
Wheelchair use at study entry															
• No	*47*	−0.94		*44*	−2.28		*47*	−0.39		*47*	−0.98		*43*	−0.99	
• Yes	*18*	−2.36		*11*	−3.92		*15*	1.44		*12*	−1.22		*11*	−2.57	
MRC / HHD sum score at study entry ^b^															
• < 33rd percentile	*18*	−1.27		*10*	−2.06		*20*	0.49		*12*	−0.30		*9*	−1.59	
• 33rd to 66th percentile	*23*	−1.31		*22*	−4.34		*23*	−0.06		*23*	−2.40		*21*	−2.01	
• ≥ 66th percentile	*24*	−0.98		*23*	−1.56		*19*	−0.41		*24*	−0.34		*24*	−0.63	

*MRC*: Medical Research Council; *HHD*: Hand-Held Dynamometry; *QMFT*: Quick Motor Function Test; *FVC*: Forced Vital Capacity; *CI*: confidence interval. ^a^ the median was taken as the cut-off point. ^b^ categorization in tertiles. *n* represents the number of patients for whom data were available for each specific measurement. For muscle strength, no sumscore was calculated if measurements for three or more individual muscle groups were missing (e.g. due to severity of muscle weakness or injury). For seven invasively ventilated patients no reliable measurements of FVC could be performed. Neither could testing in the supine position be endured by 5 patients whose pulmonary function was already severely restricted in sitting position.

Data shown are mean annual changes (percentage points per year) as calculated by repeated measures ANOVA. For MRC sumscore and HHD sumscore there were significant differences between groups based on disease duration and FVC at study entry: c) p=0.04, d) p=0.01, e) p<0.01, and f) p<0.01.

##### Pulmonary function

**Figure 5:**
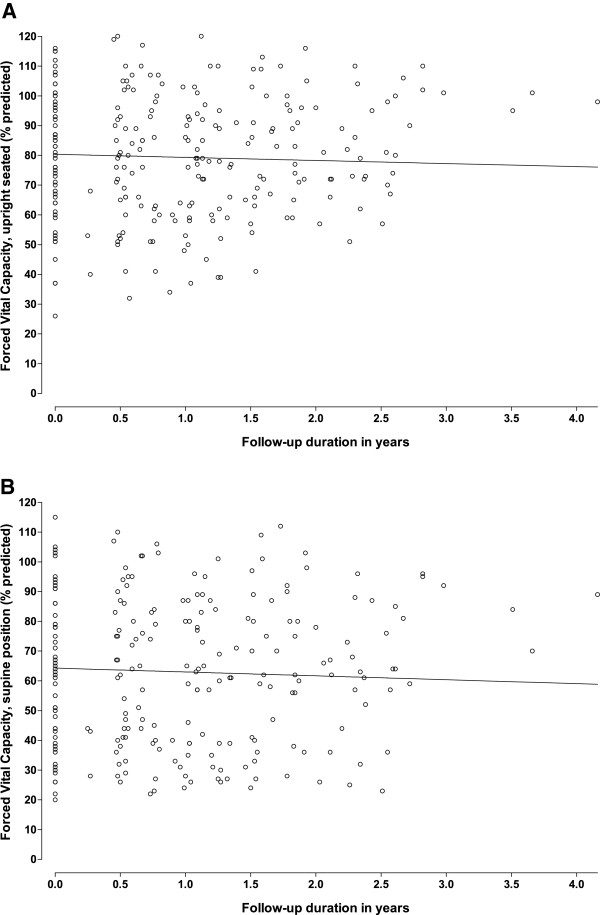
**Longitudinal changes in pulmonary function.** Decline in pulmonary function in the upright seated (**A**) and supine (**B**) positions related to follow-up duration. Circles represent the measured values, the line represents the mean regression line at a group level.

At baseline, FVC measured in sitting position was reduced (<80% of the predicted value) in 56 patients (60%). The reduction in FVC was more prominent in males than in females (mean FVC 57.5% predicted against 80.3% predicted, p<0.001). Patients with scapular winging had significantly lower FVC than those without scapular winging (mean FVC 50.7% predicted against 84.5% predicted, p<0.001). In supine position, 76 patients (80%) had a reduced FVC. Changing from a sitting to a supine position, FVC fell in 21 patients by over 25%, indicating possible diaphragmatic weakness. Neither was testing in the supine position attempted in 12 patients whose pulmonary function was already severely restricted in sitting position. We have recently reported more detailed data on pulmonary function in some of the study cohort. [23] The mean yearly change in FVC measured in supine position was 1.3% points (p=0.02), and for FVC in upright position −1.0% points (p=0.06)(Figure 5). The rate at which pulmonary function declined was consistent across subgroups. There was no significant association between the change in muscle strength or pulmonary function and residual enzyme activity (Spearman’s rho for MRC sumscore −0.21, p=0.14; for HHD sumscore −0.51, p=0.74; for FVC in upright position 0.07, p=0.65; and for FVC in supine position −0.32, p=0.84). 

#### Disease course variability

In 59 patients progress of muscle weakness and pulmonary dysfunction could be compared. In nine patients (15%; 4 males, 5 females), pulmonary function and muscle strength did not decline during the prospective follow-up period. Relative to the total group, they had a shorter disease duration from onset of symptoms (7.3 years against 15.5 years, p=0.03). At baseline, the following had all been higher, though not significantly: FVC in sitting position (88.1% against 78.6%); FVC in supine position (80.4% against 61.1%); MRC sumscore (87.2% against 83.4%); and HHD sumscore (86.2% against 76.3%). In 28 patients (47%), pulmonary function and skeletal muscle strength declined at a similar rate. In 22 patients (37%) the course of pulmonary function and muscle weakness followed different courses, one deteriorating while the other remained stable, or one deteriorating faster than the other. Of the 50 patients who deteriorated during the follow-up period, eight (2 males, 6 females) had a relatively fast decline – more than 5 percentage points per year – in muscle strength, while a rapid decline in pulmonary function was seen in five patients (1 male, 4 females). There were no significant differences between these groups with regard to sex, age, age at onset of symptoms, duration of disease, length of prospective follow-up, level of residual enzyme activity, and the severity of pulmonary or skeletal muscle involvement at the start of the study.

## Discussion

### Characteristic clinical features and pattern of muscle weakness

Our study in 94 adults with Pompe disease – which included virtually all known adult patients in the Netherlands – shows that, generally speaking, muscle weakness typically fits a pattern of limb-girdle myopathy. Interestingly, our findings about the distribution of muscle weakness, based on clinical examinations, match those of CT and MRI studies. [24-26] We found weakness of the quadriceps muscle in 55% of patients only. This may have been due to our method of measuring muscle strength: whereas the advantage of the MRC score and HHD lie in the fact that they are easy for the clinician to use, quantitative muscle testing using QMT [13,27] may be more reliable in patients with only minor loss of strength.

Besides limb-girdle weakness, many patients had less familiar features, which are sometimes symptomatic of other neuromuscular diseases. Though ptosis has been reported in the literature in no more than seven adult patients to date [28-31], it was found in almost one quarter of our patients. Notably, while one might expect myogenic ptosis to be bilateral, it was unilateral in two-thirds of our patients.

Despite the fact that bulbar muscle weakness is reported only occasionally in adult Pompe patients [28,32], twenty-eight percent of the patients in our cohort had distinct bulbar muscle weakness. These patients are at risk of pulmonary complications.

One third of patients had prominent scapular winging, which is in line with several recent reports [32,33]. Many of these patients also had bulbar muscle involvement and severer weakness of the shoulder-girdle muscles than patients without scapular winging; these features are reminiscent of FSHD. If patients with such a “pseudo-FSHD” phenotype are overlooked, diagnosis may be delayed. However, the asymmetrical distribution of muscle weakness, the relatively common involvement of the distal muscles of the lower limbs, and the absence of severe or relatively severe pulmonary involvement in FSHD will generally allow the two diseases to be diagnosed correctly [34].

Serum CK activity was normal in 10 patients, confirming that a normal serum CK does not rule out Pompe disease [35-37]. In early stages of the disease, however, normal serum CK is quite uncommon. Although we did not systematically perform electromyography or muscle biopsies, it is well known that these may reveal no or only non-specific abnormalities in a considerable number of patients [38,39]. Therefore, measurement of acid α-glucosidase activity and mutation analysis remain essential to confirm the diagnosis.

### Natural disease course

Although the average follow-up duration of 19 months seems rather short for a chronic disorder such as Pompe disease, it is the longest prospective follow-up ever carried out in “late-onset” Pompe disease. Moreover, as many patients are now treated with enzyme replacement therapy, prospective data on the natural course of the disease over a longer period of time are now impossible to obtain.

We found a significant worsening of muscle strength, reflected by declines in MRC sumscore (mean decline −1.3% pp/y) and HHD sumscore (mean decline −2.6% pp/y). Although we found significant changes in muscle strength and some patients became wheelchair or ventilator dependent during follow-up, the QMFT did not indicate a deterioration in limb-girdle muscle function. There may be two main reasons for this. First, the decline in muscle strength may have been too small to cause changes in functional daily activities within the observed time-span. Second, although the QMFT showed a good discriminative ability and good responsiveness to change after start of enzyme replacement therapy, it may not be sensitive enough to reflect minor changes in strength in functional changes during the natural course [18].

Mechanical ventilation became necessary in four of the 67 hitherto un-ventilated patients, eight patients needed to increase their number of hours of ventilation, and one patient died due to respiratory complications. Despite this indication that pulmonary function clearly worsened during the time-span of the study, our findings regarding the yearly decline seem somewhat lower than those of other studies. [13,27] This may have been due to our method of patient selection: in the present study participation was open to all adult patients with a confirmed diagnosis of Pompe disease, including those with very limited pulmonary function who were invasively ventilated 24 hours a day. Although this reflects the spectrum of disease encountered in daily practice, one would expect further deterioration of pulmonary function in these patients to be minimal, thereby partly obscuring decline in the total group. Secondly, when enzyme replacement therapy became commercially available, we decided first to treat the most severely affected patients and patients with a rapid decline in pulmonary function and muscle strength, while patients with a slow disease course were started on ERT at a later stage. As a result, the length of prospective follow-up for the most severely affected patients was somewhat shorter (1.1 years on average) than for the least affected patients (1.7 years on average, the longest follow-up being 4.2 years). Another factor that could have influenced the estimated rate of decline is the fact that data on seven patients who participated in the placebo arm of the randomized controlled trial on the safety and efficacy of alglucosidase alfa in late-onset Pompe disease were included in the present analyses. However, a second analysis, excluding these patients to overcome a possible placebo effect, led to similar estimates of the rate at which muscle weakness and pulmonary dysfunction progress (not shown).

It should be noted that all patients except one had the c.-32-13T>G (IVS1-13T>G) mutation in combination with a null allele. Although this is the most common genotype in adult Pompe patients – present in 68-93% of patients [27,40,41] – the estimated rate of disease progression may not apply to patients with different genotypes.

### Predictors for disease outcome and disease course variability

Longer disease duration (≥ 15 years) and pulmonary dysfunction (FVC <80%) were shown to be associated with a faster decline in muscle strength. Our results thus support those of the only other prospective study in adult Pompe patients, which found baseline status and duration of illness to be the most important predictors of disease severity and disease progress [27].

A subset of patients with shorter average disease duration and better baseline status did not deteriorate during follow-up, indicating that there might be a stable phase of several years before a gradual decline inevitably occurs. This raises an interesting dilemma regarding the best time to start ERT. [42] On the one hand, in patients who are only mildly affected and whose condition is stable, one might advocate that this – costly – lifelong treatment be postponed. On the other hand, studies measuring the effect of ERT show a trend toward better outcome in patients who were in a relatively good condition at the start of treatment. [11,13,14] On the basis of our results, we suggest to start ERT in all patients with pulmonary dysfunction and in patients with progressive muscle weakness, whereas in patients with minimal weakness, or in patients with solely elevated laboratory parameters treatment may be postponed, provided that they are monitored regularly. At our center, all patients undergo evaluation of muscle strength, pulmonary function, cardiac function, and hearing at referral, and evaluation of progression of muscle weakness and pulmonary dysfunction every three months thereafter.

## Conclusions

In summary, since the approval of enzyme replacement therapy in Pompe disease, early recognition of affected individuals has gained importance. The typical limb-girdle type muscle weakness – including prominent involvement of the trunk musculature – combined with early and disproportionate pulmonary involvement relative to the degree of skeletal muscle weakness should raise the suspicion of Pompe disease. Although these are the most salient characteristics, less familiar features such as ptosis, scapular winging and bulbar weakness are far more common than generally thought. If these are recognized properly, timely diagnosis will be facilitated. Longer disease duration and reduced pulmonary function stand out as the most important factors for a rapid decline in muscle strength, which may aid in deciding whom to treat and when.

## Abbreviations

4-MU: 4-methylumbelliferyl-α-D-glucopyranoside; ATS: American Thoracic Society; CCMO: Central Committee on Research Involving Human Subjects in the Netherlands; CK: Creatine Kinase; ERS: European Respiratory Society; ERT: Enzyme Replacement Therapy; FSHD: FacioScapuloHumeral Dystrophy; FVC: Forced Vital Capacity; GAA: Gene coding for acid alpha-glucosidase; HHD: Hand-held dynamometry; LGMD: Limb-Girdle Muscular Dystrophy; MRC: Medical Research Council; Pp/y: Percentage points per year; QMFT: Quick Motor Function Test; QMT: Quantitative Muscle Testing.

## Competing interests

MLCH, ATvdP, and AJJR have provided consulting services to, and have received research funding from Genzyme Corporation, a Sanofi company, under an agreement between Genzyme and Erasmus MC University Medical Center, Rotterdam, the Netherlands.The other authors declare that they have no competing interests.

## Authors’ contributions

NAMEvdB participated in study design, recruitment of patients, data collection, statistical analyses, data interpretation, and drafting and revising the manuscript for important intellectual content. JMdV participated in recruitment of patients, data collection, statistical analyses, data interpretation, and drafting and revising the manuscript for important intellectual content. MLCH participated in study design, data interpretation, and revising the manuscript for important intellectual content. WCJH participated in study design, supervised the statistical analysis, and revising the manuscript for important intellectual content. MAK participated in data collection, data interpretation, and revising manuscript for important intellectual content. JHJW, MdV, BGMvE, JBMK, AJvdK, NCN, CGF, and JJGMV participated in recruitment of patients, data interpretation, and revising the manuscript for important intellectual content. AJJR, ATvdP and PAvD conceived of the study and participated in its design and coordination, data interpretation, and revising manuscript for important intellectual content. All authors read and approved the final manuscript.

## Funding

Funding was obtained from the Erasmus MC Revolving Fund [Project No. 1054 to NAMEvdB]; ZonMw – The Netherlands Organisation for Health Research and Development [Project No. 152001005]; the Dutch TI Pharma initiative “Sustainable Orphan Drug Development through Registries and Monitoring” [Project No. T6-208]; European Union, 7^th^ Framework Programme “EUCLYD” – a European Consortium for Lysosomal Storage Diseases [health F2/2008 grant agreement 201678]; and the Prinses Beatrix Fonds [Project No. OP07-08].
